# Identification of the *StPIFs* Gene Family in Potato and Functional Analysis of *StPIF4* Under Drought Stress

**DOI:** 10.3390/plants15111623

**Published:** 2026-05-26

**Authors:** Xiangdong Wang, Tianyuan Qin, Yihao Wang, Zhuanfang Pu, Panfeng Yao, Han Wang, Yuhui Liu, Zhen Liu, Jiangping Bai, Zhenzhen Bi, Chao Sun

**Affiliations:** 1 College of Agronomy/State Key Laboratory of Aridland Crop Science, Gansu Agricultural University, Lanzhou 730070, China; wangxiangd@st.gsau.edu.cn (X.W.);; 2 Seed Industry Research Institute of Gansu Provincial University, Gansu Agricultural University, Lanzhou 730070, China; 3 Food Crops Research Institute, Xinjiang Academy of Agricultural Sciences, Urumqi 830091, China; 4 Jinchang Agricultural Research Institute, Jinchang 737100, China

**Keywords:** potato, *StPIFs*, mannitol-induced water deficit, *StPIF4* functional analysis

## Abstract

Phytochrome-interacting factors (PIF*s*) were initially recognized as pivotal regulators of plant light signaling pathways. However, mounting evidence suggests that *PIFs* also exert significant influences on plant development and responses to stress. Here, we identified seven *PIF* genes in the potato genome and conducted comprehensive characterizations through phylogenetics, gene structure, conserved motif, synteny, chromosomal location analyses and cis-regulatory element. Transcriptome data and gene expression analysis showed that the *StPIF4* gene was markedly induced by mannitol-induced water deficit. Additionally, the StPIF4 protein was primarily localized in the nucleus and plasma membrane. In order to explore the function of the *StPIF4* gene under mannitol-induced water deficit, the *StPIF4* gene was cloned, and several *StPIF4* overexpression (OE) lines (OE-8, OE-10, and OE-11) and three RNA interference (RNAi) transgenic lines (RNAi-5, RNAi-9, and RNAi-11) were obtained. The OE lines displayed notable enhancements in various growth parameters such as plant height, leaf number, branch number, fresh weight, dry weight, total root length, root surface area, number of root forks, and number of root tips under mannitol-induced water deficit compared to the wild-type (WT) lines, whereas these parameters were significantly decreased in the RNAi lines. The activities of antioxidant enzymes (SOD, POD, CAT) and the accumulation of proline and soluble sugars were also significantly increased under mannitol-induced water deficit, whereas the levels of thiobarbituric acid reactive substances (TBARSs) and reactive oxygen species (ROS), including hydrogen peroxide (H_2_O_2_) and O_2_^−^, were significantly reduced in the OE lines compared to WT plants under mannitol-induced water deficit. Moreover, the stomatal aperture of the leaves and the water loss rate in the leaves of the OE lines were significantly reduced under mannitol-induced water deficit compared to the WT plants, whereas for the RNAi lines they were significantly increased. In addition, the overexpression of *StPIF4* also upregulated expression of drought-responsive genes and ABA content under mannitol-induced water deficit. Collectively, these results highlight the positive role of the *StPIF4* gene in enhancing potato tolerance to mannitol-induced water deficit by decreasing stomatal aperture, enhancing ROS scavenging and mitigating oxidative damage.

## 1. Introduction

Ongoing climate change has rendered water deficit a critical limiting factor for plant growth [1,2]. This stress directly results in decreased plant height and can inhibit lateral root development under severe conditions; however, moderate water deficit may also stimulate primary root elongation as an adaptive strategy to access deeper soil moisture [3]. Moreover, water deficit undermines photosynthetic efficiency, induces excessive accumulation of reactive oxygen species (ROS), and exacerbates membrane lipid peroxidation [4,5]. These processes lead to increased levels of thiobarbituric acid reactive substances (TBARSs), which are associated with cellular damage and growth arrest. Ultimately, these effects culminate in diminished crop yields.

Phytochrome-interacting factors (PIFs) represent crucial constituents of the basic helix-loop-helix (bHLH) transcription factor superfamily [6]. PIF proteins exhibit three conserved structural elements: an N-terminal bHLH domain accountable for DNA binding, APB/APA motifs facilitating phytochrome interactions, and a C-terminal transcriptional activation domain [7]. To date, *PIF* family members have been detected in various plant species such as Arabidopsis [8], rice [9], corn [10], wheat [11], tomato [12], and grape [13]. The function of PIF proteins has been extensively studied across various plant species. In Arabidopsis, the *PIF* gene family is essential for mediating photomorphogenesis, thermomorphogenesis, and adaptation to water deficit [9,14]. Similarly, the rice OsPIF proteins demonstrate considerable evolutionary conservation and can be categorized into three primary branches, with their expression levels being significantly influenced by osmotic and salt stress [15]. Furthermore, all members of the soybean *GmPIF* gene family possess a complete bHLH DNA-binding domain, and promoter analysis has indicated the presence of cis-acting elements that respond to drought, high temperature, shading, and plant hormones [16].

Accumulating evidence indicates that *PIF* genes are crucial in plants’ responses to abiotic stress [6,17]. For instance, overexpression of *AtPIF3* enhances tolerance to water deficit by reducing transpiration efficiency. *DcPIF3* inhibits mannitol-induced water deficit-induced reactive oxygen species (ROS) burst and enhances the expression levels of genes associated with ABA synthesis, leading to an increased content of endogenous ABA and promoting the tolerance to mannitol-induced water deficit in carrot (*Daucus carota* L.). *AtPIF4* improves heat tolerance in Arabidopsis by suppressing the expression of the downstream *SPCH* gene [18]. *ZmPIF1* increases tolerance to water deficit and yield in maize by inducing stomatal closure [19]. *OsPIL16* positively regulates cold tolerance by binding to the promoter region of the *DREB* gene and activating its transcription [20].

Potato (*Solanum tuberosum* L.) is a drought-sensitive crop; drought stress impairs plant growth, development, and physiology, ultimately leading to yield reduction [21]. Therefore, it is imperative to identify crucial drought-resistance genes and understand their molecular regulatory mechanisms to enhance tolerance to water deficit tolerance. While several drought-responsive genes have been characterized in potato, such as *StNAC053* [22], *StWRKY75* [23], *StbHLH47* [24], *StNCED2* [25], *StDHN1* [26], *StCDPK13* [27], and *StJAZ23* [28], the functional role of *PIF* genes in response to mannitol-induced water deficit remains unexplored in potato. However, in this study, seven *StPIF* genes were identified in potato and subjected to phylogenetic, gene structure, motif, and chromosomal location analyses. The *StPIF* genes’ expression profiles were also analyzed, and the *StPIF4* gene was selected to undergo detailed functional analysis under mannitol-induced water deficit. Then, the *StPIF4* gene was cloned, and the transgenic overexpression (OE) and RNA interference (RNAi) in potato plants were obtained. The phenotypes, physiological indicators, leaf stomatal aperture, leaf relative water loss rate, and gene expression were analyzed in the OE lines and RNAi lines under drought stress to identify the function of *StPIF4* under mannitol-induced water deficit. These findings establish a molecular framework for understanding PIF4-mediated mannitol-induced water deficit responses in tuber crops and provide valuable genetic resources for the breeding of climate-resilient potato varieties.

## 2. Results

### 2.1. Identification of StPIF Genes

Seven *StPIFs* were identified in the Spud DB potato genome and named *StPIF1* to *StPIF7*, distributed across the 12 potato chromosomes (Figure S1). Analysis of the physicochemical properties of the StPIF proteins showed amino acid sequence lengths ranging from 418 to 636 residues, with an average of 502 residues and molecular weights ranging from 46.9 to 68.5 kDa, averaging 54.91 kDa. The theoretical isoelectric points (pI) ranged from 4.91 to 8.80, with most proteins exhibiting an alkaline nature (pI > 7). Subcellular localization prediction indicated that all StPIF proteins are localized in the nucleus (Table S1).

### 2.2. Phylogenetic Analysis

**Figure 1:**
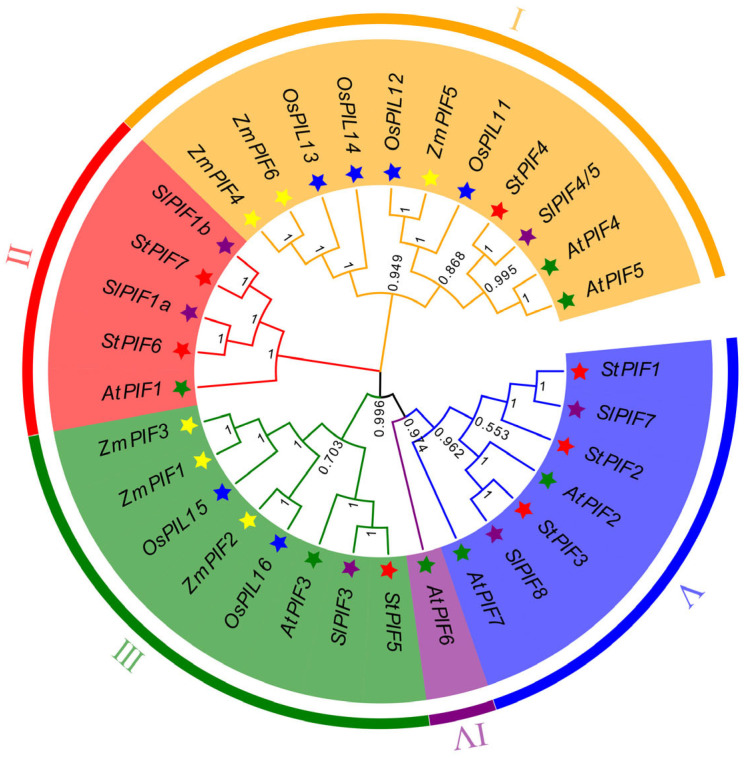
The neighbor-joining phylogenetic tree of PIF proteins in potato, Arabidopsis, tomato, rice, and maize. The 32 PIFs were divided into 5 groups: orange, red, green, purple, and blue. Green stars, red stars, purple stars, blue stars, and yellow stars represent Arabidopsis, potato, tomato, rice, and maize. The phylogenetic tree was constructed using MEGA software (MEGA 7.0) (with the Maximum Likelihood (ML) method and 1000 bootstrap replicates).

Phylogenetic analysis included PIF proteins from potato, Arabidopsis thaliana, rice, tomato, and maize, resulting in a tree with five distinct groups (Groups I–V) (Figure 1). Among them, the *PIF* genes of potato, rice, tomato, and maize are divided into four subfamilies. In the fifth subgroup, there is only one *PIF* gene in Arabidopsis. Close clustering of several StPIF proteins with *SlPIFs* suggested high sequence similarity, indicative of conserved evolutionary relationships and potential functional similarity.

### 2.3. Gene Structure and Motif Analyses

**Figure 2:**
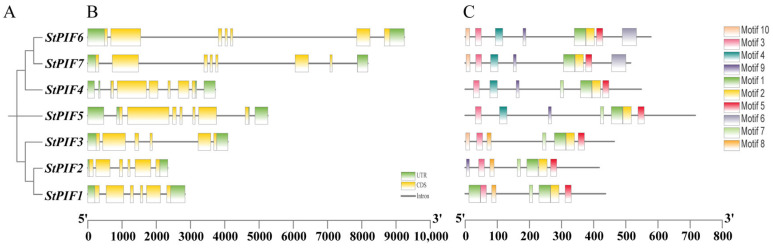
Structural analysis of potato *StPIFs*. (**A**) Phylogenetic tree constructed from seven StPIF proteins using MEGA 7 (Maximum Likelihood method, 1000 bootstrap replicates). (**B**) Gene structures of seven *StPIF* genes visualized using TBtools (v2.467) based on the potato genome annotation. (**C**) Composition and distribution of conserved motifs of StPIF proteins identified using the MEME Suite (v5.4.1) with the following parameters: maximum number of motifs = 10; minimum width = 6; maximum width = 50.

Conserved motifs in the seven potato StPIF proteins were identified through the MEME program, leading to the recognition of 10 distinct motifs (Figure 2C). Examination of motif distribution revealed that each StPIF protein harbored between seven and eight motifs. All seven StPIF proteins shared motifs 1, 2, 3, and 5. Motif 7 was present in StPIF1, StPIF2, StPIF3, StPIF4, and StPIF5, while motif 6 was exclusively found in StPIF6 and StPIF7. Furthermore, motif 10 was identified in StPIF3, StPIF6, and StPIF7, whereas motif 8 was specific to StPIF1, StPIF2, and StPIF3. These findings underscore the functional significance and conserved arrangement of motifs in StPIF proteins. Moreover, most members of the StPIF group in the same phylogenetic cluster displayed comparable motif compositions, indicating potential functional resemblance. Evolutionary divergence in gene families often coincides with variations in exon–intron arrangement. Analysis of gene structure revealed that *StPIF* genes shared akin exon–intron architectures, typically comprising six or seven exons and four to eight introns (Figure 2B). Among *StPIF* gene family members, three (*StPIF5*, *StPIF6*, and *StPIF7*) featured seven exons, while four (*StPIF1*, *StPIF2*, *StPIF3*, and *StPIF4*) had six exons. All *StPIF* genes consisted of multiple exons. Genes belonging to the same phylogenetic cluster commonly displayed analogous gene structures, encompassing similar exon count, exon length, and overall gene length.

### 2.4. Analysis of the StPIF Genes for Gene Collinearity

**Figure 3:**
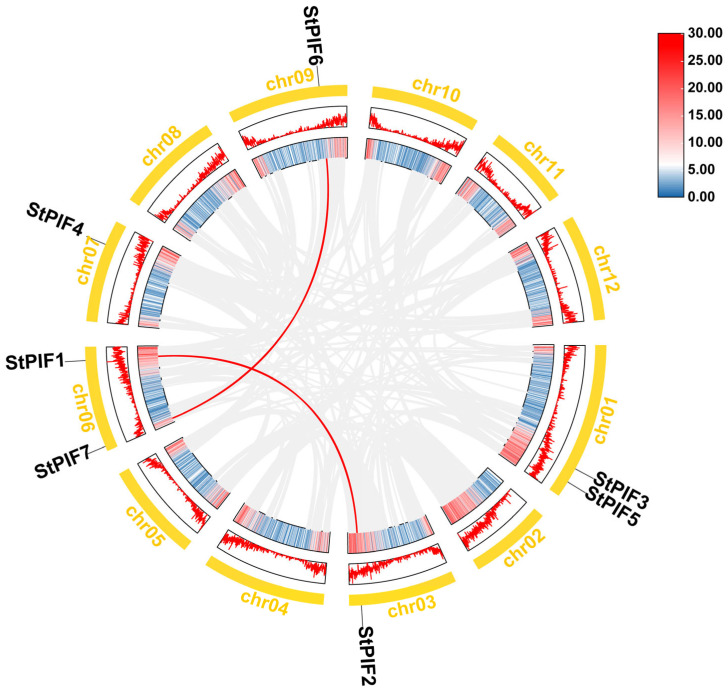
Synteny distribution of *StPIF* genes in potato. Red lines connect collinear gene pairs. Synteny analysis was performed using the Multiple Collinearity Scan toolkit (MCScanX) implemented in TBtools (v2.467) with default parameters.

**Figure 4:**
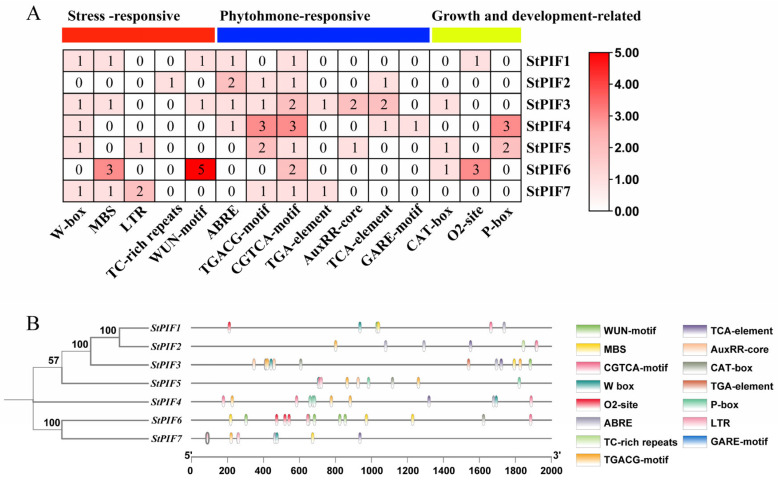
Analysis of cis-acting elements in *StPIFs*. Analysis of cis-acting elements was conducted on 2 kb sequences upstream of coding sequences of *StPIFs*. (**A**) Classification and number of cis-acting elements on the promoter region of StPIFs. (**B**) Distribution of cis-acting elements in promoter region of *StPIFs*. Cis-acting elements were predicted using the PlantCARE database (http://bioinformatics.psb.ugent.be/webtools/plantcare/html/, accessed on 4 February 2025) and visualized using TBtools (v2.467).

During evolution, gene family expansion is primarily driven by tandem duplication and large-scale duplication events. These duplication events increase genomic complexity and facilitate functional diversification of genes. Synteny analysis of the potato *StPIF* gene family revealed the presence of two segmentally duplicated gene pairs, with no tandem duplication events detected (Figure 3). Specifically, *StPIF1* was segmentally duplicated with *StPIF2*, and *StPIF6* formed another segmentally duplicated pair with *StPIF7* (Figure 4). These results suggest that segmental duplication has played a role in the evolution of the *StPIF* gene family.

### 2.5. Analysis of Promoter Cis-Acting Elements of the StPIFs Genes

Analysis of cis-acting elements in the promoter regions indicated that these elements were primarily associated with stress responses, phytohormone signaling, and the regulation of growth and development (Figure 4B). Among the stress-responsive cis-acting elements, the W-box was the most prevalent, appearing in five *StPIF* genes. The drought-responsive MBS element was identified in four *StPIF* genes. Additionally, the low-temperature-responsive element (LTR) was found in two *StPIF* genes, while TC-rich repeats were detected in one *StPIF* gene. Several hormone-responsive cis-acting elements were also identified; notably, the abscisic acid-responsive element (ABRE) was present in four *StPIF* genes. The jasmonic acid-responsive TGACG-motif was identified in five *StPIF* genes, exhibiting a notably higher frequency in *StPIF4*. The CGTCA motif, another element responsive to methyl jasmonic acid, was present in all seven *StPIF* genes, with the highest enrichment observed in *StPIF4*. Additionally, the auxin-responsive TGA element was found in *StPIF3* and *StPIF7*, while the cytokinin-responsive AuxRR-core element was detected in three *StPIF* genes. Among the cis-acting elements related to growth and development, the CAT-box was present in three *StPIF* genes. The O_2_^−^ site, a regulatory element linked to endosperm expression, was identified in two *StPIF* genes, predominantly in *StPIF6*. The P-box was also recognized in two *StPIF* genes, with a greater occurrence in *StPIF4*. Collectively, the composition of cis-elements suggests potential functional differentiation among *StPIF* genes concerning stress responses, hormone signaling, and growth and development (Figure 4A).

### 2.6. Expression Patterns Analysis of StPIFs Genes in Different Tissues and in Response to Abiotic Stresses

**Figure 5:**
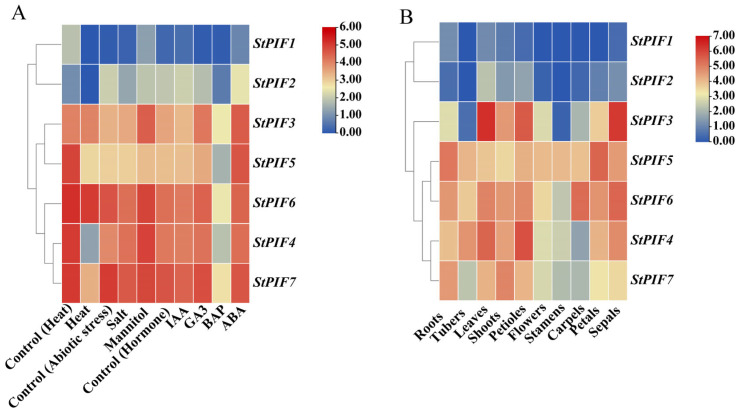
Expression patterns of seven *StPIF* genes in potato. (**A**) Expression profiles of *StPIF* genes in different tissues and organs. (**B**) Expression profiles of *StPIF* genes under different stress treatments. RNA-seq expression data were retrieved from the International Potato Genome Sequencing Consortium (PGSC) dataset for further analysis. Heat maps were generated using TBtools (v2.467) based on log2-transformed RPKM (reads per kilobase of exon model per million mapped reads) values.

The expression patterns of seven *StPIFs* genes across various potato tissues, including roots, tubers, leaves, shoots, petioles, flowers, stamens, carpels, petals, and sepals, were examined using RNA-seq data. The analysis revealed significant differences in the expression patterns of *StPIFs* genes among the different tissues (Figure 5A). Notably, *StPIF1* and *StPIF2* (Group V) exhibited consistently low expression levels across all tissues. In contrast, *StPIF3* (Group V) demonstrated relatively low expression in tubers and stamens, while showing high expression in other tissues, indicating distinct tissue specificity. Members of Groups II and III generally displayed higher transcript abundance across multiple tissues, particularly *StPIF4* and *StPIF6*. Additionally, the expression profiles of *StPIF* genes under abiotic stress conditions were assessed using RNA-seq data (Figure 5B). Compared to the control, *StPIF3* and *StPIF4* exhibited strong induction under mannitol and ABA treatments. To confirm the RNA-seq findings, the expression of seven *StPIF* genes was further assessed via qPCR in potato exposed to mannitol and ABA stresses. The results showed that the expression of the *StPIF4* gene was significantly induced under mannitol-induced water deficit and ABA treatment and increased by 8-fold and 25-fold at 12 h and 24 h after treatment, respectively.

**Figure 6:**
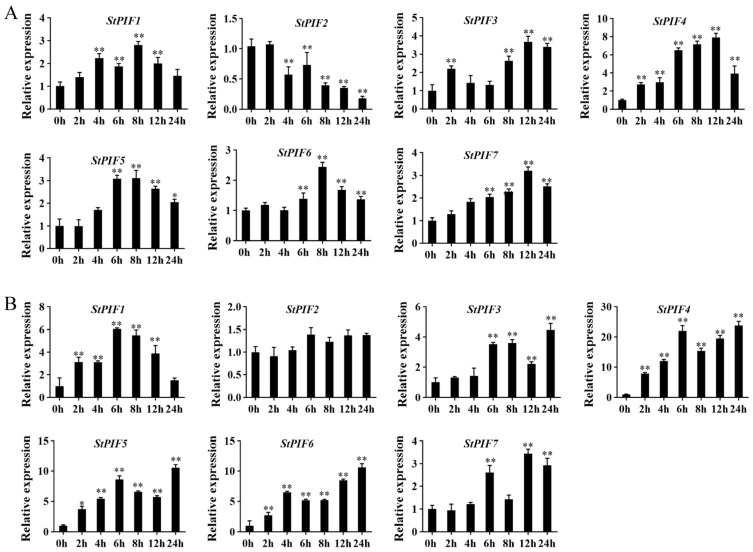
Expression profiles of *StPIF* genes following different treatments. (**A**) Mannitol, (**B**) ABA. The transcript levels of each *StPIF* gene in the stress-treated plants (0, 2, 4, 6, 8, 12 and 24 h treatment) were plotted as the relative expression of the untreated control plants. (*n* = 3; for each biological replicate, three technical replicates were performed. Data represent the means ± SD (standard deviation) of three replicates. * and ** indicate significant differences at *p* < 0.05 and *p* < 0.01 levels.

Upon mannitol treatment, *StPIF3*, *StPIF4*, *StPIF6*, and *StPIF7* were significantly upregulated (Figure 6A). ABA treatment triggered the upregulation of *StPIF3*, *StPIF4*, *StPIF5*, *StPIF6*, and *StPIF7*, with *StPIF4* and *StPIF5* displaying the most prominent upregulation (Figure 6B). Particularly noteworthy is the robust induction of *StPIF4* expression by both mannitol and ABA treatments, peaking at 24 h.

### 2.7. Generation of Stable Transgenic Potato Plants

**Figure 7:**
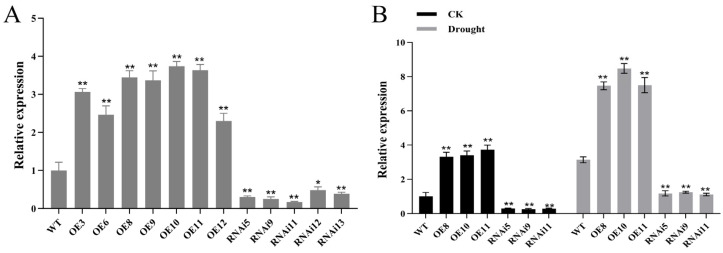
Expression levels of the *StPIF4* gene. (**A**) Expression level of *StPIF4* in OE and RNAi lines under control conditions. (**B**) Relative expression of *StPIF4* under normal treatment (CK: 0 mM mannitol) and mannitol-induced water deficit (drought: 150 mM mannitol). Data represent the means ± SD (standard deviation) of three replicates. * and ** Indicates significant differences in the OE line and RNAi line compared with WT (* *p* < 0.05 and ** *p* < 0.01).

To further characterize the biological function of *StPIF4* under mannitol-induced water deficit, OE and RNAi vectors were constructed and introduced into potato plants (Figure S2A,B). Kanamycin-resistant transgenic E3 potato plants were identified by PCR and qPCR analyses (Figures S2C). The results showed that *StPIF4* expression levels varied among different transgenic lines, likely due to differences in transformation efficiency. Among the OE lines, OE-11 showed the highest expression level (3.72), followed by OE-10 (3.46) and OE-8 (3.35) relative to the wild type. Accordingly, three OE lines with the highest *StPIF4* expression levels (OE-8, OE-10, and OE-11) were selected for subsequent functional analyses. In the RNAi lines, *StPIF4* expression was markedly reduced, with RNAi-5, RNAi-9, and RNAi-11 showing relative expression levels of 0.25, 0.17, and 0.32, respectively. Therefore, OE-8, OE-10, and OE-11, together with RNAi-5, RNAi-9, and RNAi-11, were selected for subsequent functional validation experiments. Expression levels were significantly higher in OE lines and lower in RNAi lines compared to WT lines (Figure 7B). Under the drought condition (100 mM mannitol), the expression levels were increased 2.1–2.8 times in OE lines, while RNAi lines were decreased to 48.2–53.4%.

### 2.8. Organ-Specific Expression and Subcellular Localization Analysis of StPIF4

**Figure 8:**
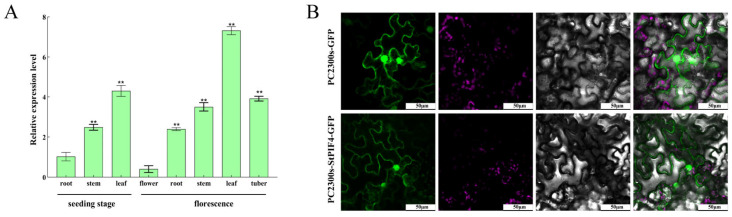
Organ-specific expression and subcellular localization analysis of *StPIF4*. (**A**) The expression level of *StPIF4* in flower, root, stem, leaf and tuber was analyzed by qPCR. (**B**) Subcellular localization of *StPIF4*. Bars = 50 µm. (Data represent the means ± SD (standard deviation) of three replicates. ** indicates significant differences at *p* < 0.05 and *p* < 0.01 levels.)

Gene expression patterns provide valuable insights into gene function. Consequently, the expression levels of *StPIF4* across various potato organs were investigated. The results showed transcript abundance was highest in leaves, followed by tubers, stems, and roots, with flowers exhibiting the lowest level (Figure 8A). Meanwhile, to ascertain the subcellular localization of the StPIF4 protein, *PC2300s-GFP* and *PC2300s-StPIF4-GFP* vectors were transferred into Agrobacterium tumefaciens GV3101 and then injected into tobacco leaves to observe the fluorescence signal. The results showed that green fluorescence derived from the *PC2300s-StPIF4-GFP* was primarily concentrated in the cell membrane and nucleus of tobacco leaf epidermal cells. In contrast, the control *PC2300s-GFP* exhibited fluorescence throughout the cell membrane, cytoplasm, and nucleus (Figure 8B).

### 2.9. StPIF4 Positively Regulating Tolerance to Mannitol-Induced Water Deficit in Potato

**Figure 9:**
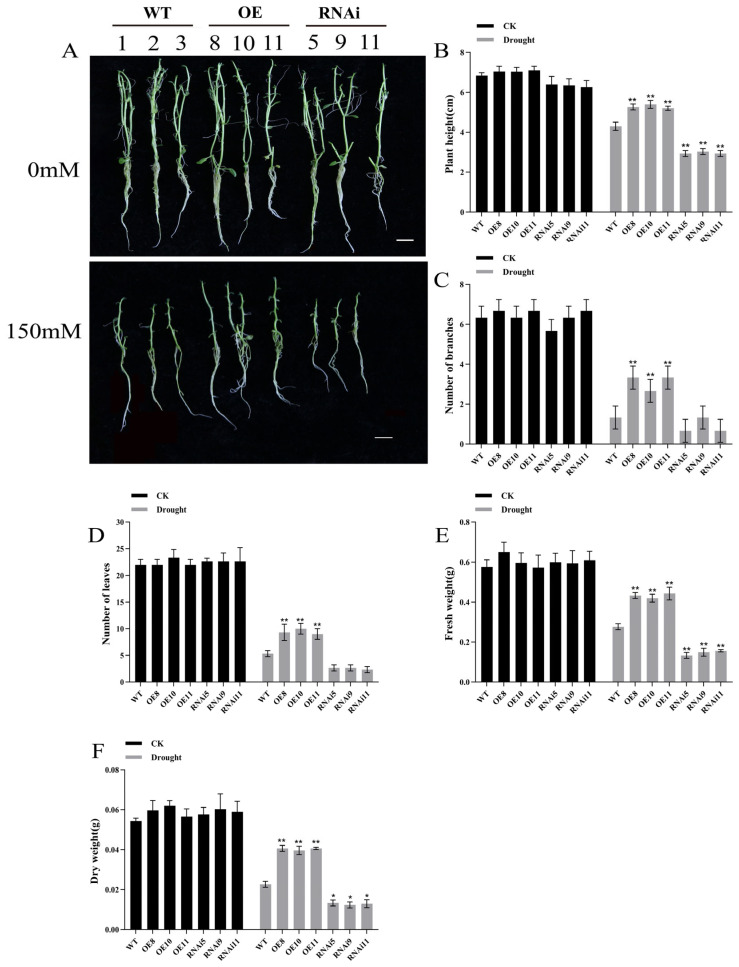
Changes in phenotypes and related parameters of *StPIF4* transgenic plants under mannitol-induced water deficit. (**A**) Phenotypes of the plants, (**B**) plant height, (**C**) number of branches, (**D**) number of leaves, (**E**) fresh weight, and (**F**) dry weight under normal treatment (CK: 0 mM mannitol) and mannitol-induced water deficit (drought: 150 mM mannitol). (n = 3 biological replicates × 3 plantlets per replicate = 9 plantlets per treatment per line.) Data represent the means ± SD (standard deviation) of three replicates. * and ** Indicates significant differences in the OE line and RNAi line compared with WT (* *p* < 0.05, ** *p* < 0.01). Bars = 2 cm.

**Figure 10:**
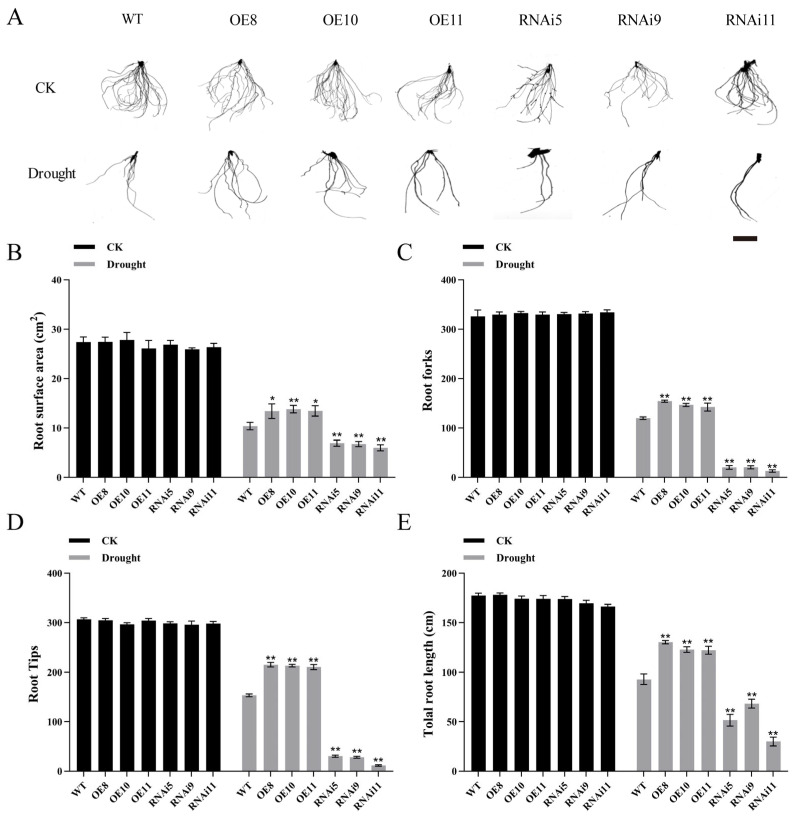
Root phenotype and related parameter changes in *StPIF4* transgenic plants under drought conditions. (**A**) Morphology of roots, (**B**) root surface area, (**C**) root forks, (**D**) root tips, and (**E**) total root length under normal treatment (CK: 0 mM mannitol) and mannitol-induced water deficit (drought: 150 mM mannitol). (n = 3 biological replicates × 3 plantlets per replicate = 9 plantlets per treatment per line.) Data represent the means ± SD (standard deviation) of three replicates. * and ** Indicates significant differences in the OE line and RNAi line compared with WT (* *p* < 0.05, ** *p* < 0.01). Bars = 2.5 cm.

To investigate the role of *StPIF4* in drought resistance, the OE lines, RNAi lines, and WT potato lines were subjected to 150 mM mannitol-simulated, mannitol-induced water deficit. Under normal conditions, no significant phenotypic differences were observed among the OE, RNAi, and WT lines. However, under mannitol-induced water deficit, the transgenic plants exhibited superior growth compared to the WT lines. After exposure to 150 mM mannitol, significant increases in plant height, leaf number, branch number, fresh weight, and dry weight were observed in OE lines relative to wild-type (WT) plants (Figure 9). In contrast, these growth parameters were markedly reduced in the RNAi lines compared to WT lines. These findings suggested that *StPIF4* positively regulates potato growth and development under mannitol-induced water deficit. Further analysis of root phenotypes and related parameters revealed that under mannitol-induced water deficit conditions, the OE lines displayed significantly greater root length, number of root branches, number of root tips, and root surface area than the WT lines. Conversely, all root-related indices in the RNAi lines were significantly lower than those in the WT lines (Figure 10). These results reinforce the conclusion that *StPIF4* positively regulates potato growth and development under mannitol-induced water deficit.

### 2.10. Overexpression of the StPIF4 Gene Inhibits ROS Accumulation and the Increase in Antioxidant Enzyme Activity in Potato Under Mannitol-Induced Water Deficit

**Figure 11:**
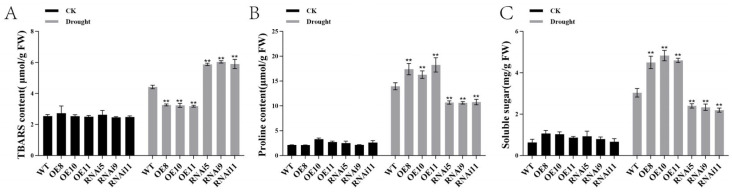
Determination of malondialdehyde, proline and soluble sugar content in WT and *StPIF4*-overexpressing and RNA interference transgenic potato plants under control and drought conditions, as well as drought resistance assessment of WT and *StPIF4*-overexpressing and RNA interference transgenic potato plants. (**A**) TBARS content, (**B**) proline content, and (**C**) soluble sugar content under normal treatment (CK: 0 mM mannitol) and mannitol-induced water deficit (drought: 150 mM mannitol). Data represent the means ± SD (standard deviation) of three replicates. ** Indicates significant differences in the OE line and RNAi line compared with WT (** *p* < 0.01).

In order to further assess the impact of mannitol-induced water deficit on oxidative damage, leaves of WT, OE, and RNAi lines were collected after mannitol-induced water deficit treatment for DAB and NBT staining and physiological analysis. Additionally, antioxidant enzyme activities were evaluated in the leaves of WT and transgenic plants. Under normal conditions, the activities of superoxide dismutase (SOD), peroxidase (POD), and catalase (CAT) did not differ significantly among the tested lines. In contrast, after mannitol-induced water deficit, the activities of SOD, POD, and CAT were significantly increased in OE lines compared to WT lines, whereas RNAi lines demonstrated significantly lower enzyme activities (Figure 11B–D). Meanwhile, H_2_O_2_ content was significantly reduced in OE lines but markedly increased in RNAi lines relative to WT lines. In addition, the NBT and DAB staining of leaves was done to detect the accumulation of O_2_^−^ and H_2_O_2_ in the OE, RNAi and WT lines.

**Figure 12:**
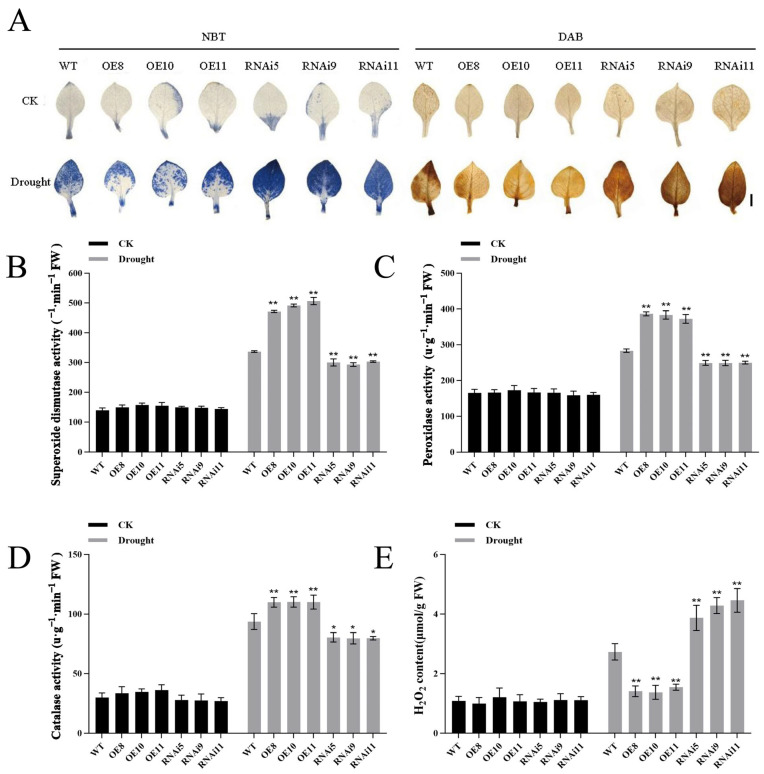
ROS accumulation and antioxidant enzyme activities in leaves of WT, *StPIF4*-overexpressing, and RNA interference transgenic potato plants. (**A**) NBT staining and DAB staining, (**B**) SOD activities, (**C**) POD activities, (**D**) CAT activities, and (**E**) H_2_O_2_ content under normal treatment (CK: 0 mM mannitol) and mannitol-induced water deficit (drought: 150 mM mannitol). Data represent the means ± SD (standard deviation) of three replicates. * and ** Indicates significant differences in the OE line and RNAi line compared with WT (* *p* < 0.05, ** *p* < 0.01). Bar = 2 mm.

Mannitol-induced water deficit can compromise plant cell membranes, and the levels of thiobarbituric acid reactive substances (TBARSs) serve as an indicator of membrane damage.Under control conditions, the TBARS content did not differ significantly among WT and transgenic plants. However, following drought stress, the TBARS content in OE lines was significantly lower than those in WT lines, while in RNAi lines it was signifi-cantly higher (Figure 11A). Proline and soluble sugars are crucial for osmotic regulation, helping to respond to and alleviate the damage inflicted by drought stress. After mannitol-induced water deficit, the contents of proline and soluble sugars in OE lines were significantly higher than those in WT lines, whereas RNAi lines exhibited significantly lower contents (Figure 11B–C). Additionally, antioxidant enzyme activities were evaluated in the leaves of WT and transgenic plants. Under normal conditions, the activities of superoxide dis-mutase (SOD), peroxidase (POD), and catalase (CAT) did not differ significantly among the tested lines. In contrast, after mannitol-induced water deficit, the activities of SOD, POD, and CAT were significantly increased in OE lines compared to WT lines, whereas RNAi lines demonstrated significantly lower enzyme activities (Figure 12B–D). Meanwhile, H_2_O_2_ content was significantly reduced in OE lines but markedly in-creased in RNAi lines relative to WT lines (Figure 12E). In addition, the NBT and DAB staining of leaves was done to detect the accumulation of O_2_^−^ and H_2_O_2_ in the OE, RNAi and WT lines. The results showed that under normal conditions, there were no significant differences in NBT and DAB staining among WT and transgenic plants (Figure 12A). However, after mannitol-induced water deficit, the staining intensity in OE lines was significantly lower than that in WT lines, while RNAi lines exhibited markedly stronger staining compared to WT lines. This observation indicates a reduction in O_2_^−^ and H_2_O_2_ accumulation in OE lines. Collectively, these findings suggest that the overexpression of *StPIF4* enhances drought tolerance by promoting ROS clearance and mitigating oxidative damage.

### 2.11. StPIF4 Regulates Stomatal Aperture and Relative Water Loss Rate of Leaves

**Figure 13:**
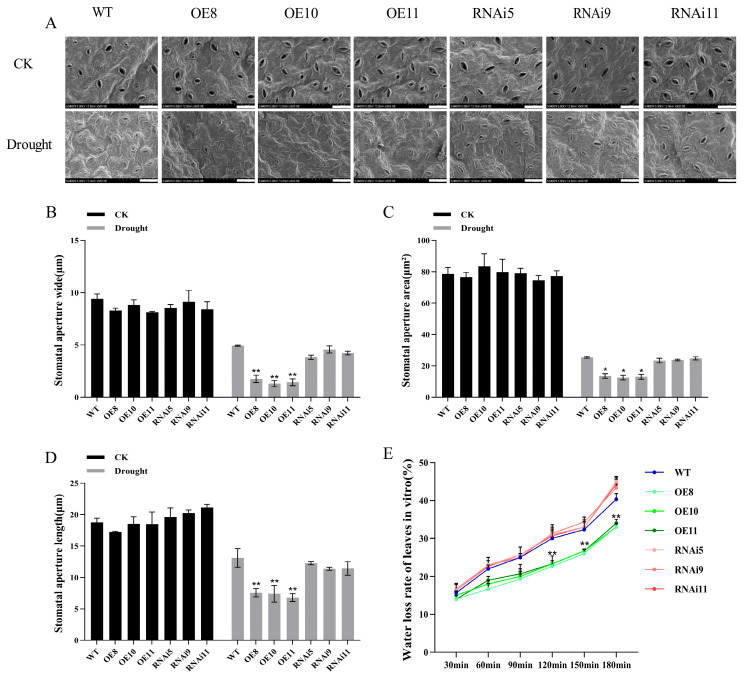
Effects of drought on leaf stomatal conductance and leaf water loss in WT, *StPIF4*-overexpressing, and RNAi transgenic potato plants. (**A**) Phenotype of stomatal phase in OE (8, 10, 11) and RNAi (5, 9, 11) and WT lines with mannitol treatment. (**B**) Stomatal aperture length, (**C**) stomatal aperture width, (**D**) stomatal aperture area, and (**E**) relative water loss rate of leaves in vitro under normal treatment (CK: 0 mM mannitol) and mannitol-induced water deficit (drought: 150 mM mannitol). Data represent the means ± SD (standard deviation) of three replicates. * and ** Indicates significant differences in the OE line and RNAi line compared with WT (* *p* < 0.05, ** *p* < 0.01). Bars = 50 µm.

**Figure 14:**
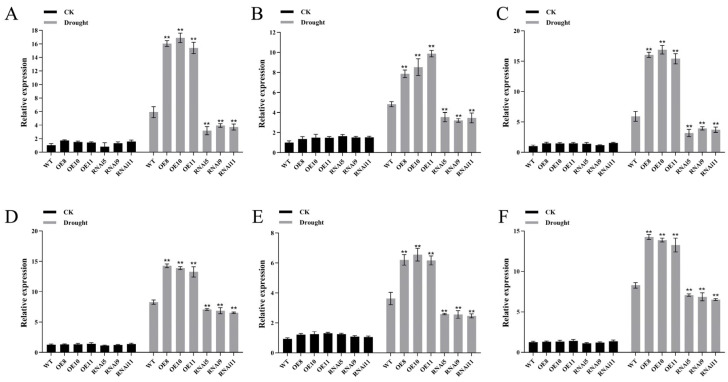
The expression levels of the six drought genes were detected by qPCR under drought stress and control conditions. (**A**) *StPGAM*, (**B**) *StDR29A*, (**C**) *StDREB*, (**D**) *StABI5,* (**E**) *StNCED3*, and (**F**) *StP5CS* under normal treatment (CK: 0 mM mannitol) and mannitol-induced water deficit (drought: 150 mM mannitol). (*n* = 3; for each biological replicate, qPCR was performed with three technical replicates.) Data represent the means ± SD (standard deviation) of three replicates. ** Indicates significant differences in the OE line and RNAi line compared with WT (** *p* < 0.01).

Leaves from WT, OE, and RNAi lines were gathered for stomatal examination following drought stress (Figure 13A). Stomatal morphology did not differ significantly between WT and transgenic plants under normal growth conditions. However, during mannitol-induced water deficit, the OE lines exhibited significantly reduced stomatal aperture and area compared to WT lines, while the RNAi lines showed no significant deviations in WT lines (Figure 13B–D). To assess water retention capability further, the water loss rate of detached leaves was also quantified. It was found that leaves from OE lines demonstrated a markedly lower water loss rate than those from WT and RNAi lines throughout the dehydration period (Figure 14E). Conversely, RNAi lines exhibited a water loss rate similar to or slightly higher than that of WT lines. These results indicate that the overexpression of *StPIF4* enhances drought tolerance in potato plants by promoting stomatal closure and decreasing water loss.

### 2.12. Expression Analysis of Drought-Responsive Genes in Potato

To further investigate the potential role of *StPIF4* in drought stress, we employed qPCR to assess the expression levels of six drought-responsive genes (phosphoglycerate mutase (*PGAM)*, desiccation-responsive protein 29A (*DR29A*), dehydration-responsive element-binding protein (*DREB)*, ABA-insensitive 5 (*ABI5*), 9-cis-epoxycarotenoid dioxygenase 3 (*NCED3*) and pyrroline-5-carboxylate synthetase (*P5CS*)) in WT, OE, and RNAi lines (Figure 14). The results indicate that, following drought stress, the expression of drought stress-related genes was significantly increased in the OE lines compared to the WT line. These findings suggest that the overexpression of *StPIF4* enhances the expression of drought response genes, thereby bolstering the plants’ capacity to endure drought conditions.

### 2.13. ABA Content Detection

**Figure 15:**
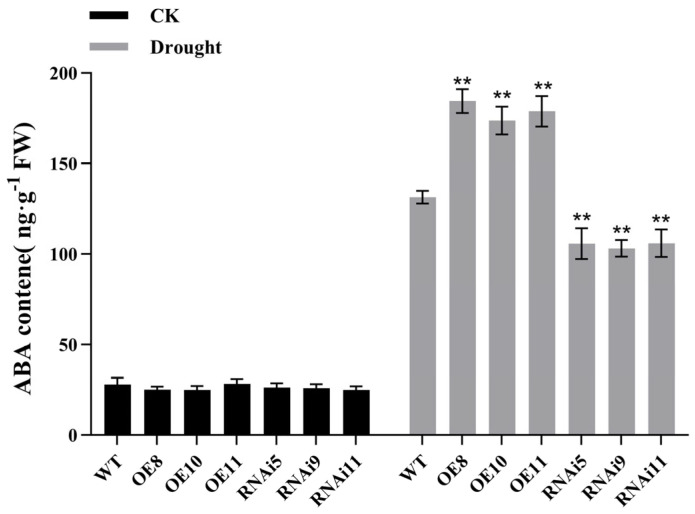
ABA content determination. CK and drought treatment conditions were applied to the plant, and random samples of leaves from different plants were collected. Quantitative analysis of ABA was performed using high-performance liquid chromatography (HPLC) under normal treatment (CK: 0 mM mannitol) and mannitol-induced water deficit (drought: 150 mM mannitol). n = 3. Data represent the means ± SD (standard deviation) of three replicates. ** Indicates significant differences in the OE line and RNAi line compared with WT (** *p* < 0.01).

The *PIF4* gene exhibits significant expression under ABA treatment. To further validate whether the *StPIF4* gene regulates ABA production, we measured ABA content in plants from different lines under various treatments (Figure 15). Experimental results indicate that under normal conditions, there are no significant changes in ABA content within the WT, OE, and RNAi lines. Under mannitol-induced water deficit, ABA content in OE lines was significantly increased compared to WT lines, whereas in RNAi lines it was markedly reduced.

## 3. Discussion

The *PIF* gene family has been identified in a variety of plant species. For instance, seven members have been recognized in Arabidopsis thaliana, while eight candidate *PIF* genes have been reported in tomato, maize and apple. In rice, six members have been identified, whereas wheat has been shown to contain seventeen members [6]. Furthermore, analysis of cis-acting elements in the promoter regions of *PIF* gene family members in Medicago sativa revealed a significant number of elements linked to plant responses to abiotic stresses [29]. In this study, seven members of the *PIF* gene family were identified from the potato genome and divided into four subfamilies. Most *StPIF* gene family members have promoter regions containing elements related to light response, plant growth and development, hormone signal transduction, and stress response. This suggests their involvement in multiple signaling pathways and response to abiotic stress.

Abiotic stress has an important impact on plant growth and development. Under drought conditions, root gravitropic growth and the ability to penetrate deep into the soil are critical determinants of a plant’s water acquisition [30]. Extensive research has demonstrated that drought stress induces significant changes in plant phenotypes. For example, the overexpression of *GmMYB14* in soybean has been shown to modulate plant height and stem diameter through the brassinosteroid (BR) signaling pathway under drought stress [31,32]. Similarly, *TCP13*, a dehydration-inducible CIN-type *TCP* transcription factor, has been reported to negatively regulate leaf and root development in Arabidopsis thaliana under drought stress, thereby altering plant architecture [33]. Notably, the overexpression of *IPT* has been shown to contribute to the maintenance of leaf number and leaf area under drought stress [34]. *ZmSNAC06* in maize and *OsNAC5* in rice are root-specific transcription factors that enhance drought tolerance by promoting both lateral root number and length, thereby increasing the root-to-shoot ratio [35]. Overexpression of *DRO1* in various crops has been shown to improve drought tolerance by significantly increasing root length and root fresh biomass, thereby enhancing the plant’s capacity to acquire soil water [36]. In this study, under mannitol-induced water deficit, OE potato plants exhibited notable increases in plant height, branch number, leaf number, fresh weight, and dry weight compared to WT plants. Further examination of root traits revealed that OE plants also displayed significant enhancements in root length, root surface area, number of lateral roots, and root tip number. These results indicate that overexpression of *StPIF4* confers improved drought tolerance in potato.

Drought stress adversely affects the plant antioxidant system, leading to a substantial accumulation of reactive oxygen species (ROS) [37]. Previous studies have shown that excessive ROS accumulation disrupts plant cell structure and compromises cellular viability [38]. To mitigate oxidative stress, plants enhance the activities of antioxidant enzymes, such as superoxide dismutase (SOD), peroxidase (POD), and catalase (CAT), thereby removing ROS accumulation [39]. In rice, *OsANN9*-overexpressing plants exhibited significantly elevated SOD, POD, and CAT activities under drought stress, with concomitant reductions in H_2_O_2_, O_2_^−^, and TBARS levels, indicating enhanced ROS scavenging and maintenance of cellular redox homeostasis [40]. Similarly, overexpression of *NtWRKY65* in tobacco significantly activated CAT, SOD, and POD activities under 150 mM NaCl treatment, concomitant with reductions in TBARS content and relative electrical conductivity, demonstrating that *NtWRKY65* alleviates salt-induced oxidative damage through the enzymatic antioxidant pathway [41]. In this study, under mannitol-induced water deficit, OE plants exhibited significantly increased levels of soluble sugars, proline, and antioxidant enzymes (SOD, POD, and CAT) compared to WT plants, along with reduced levels of H_2_O_2_ and O^2−^. Drought stress can damage the integrity of cell membranes. TBARS is the final decomposition product of membrane lipid peroxidation, and the TBARS content can reflect the extent of cell membrane damage. Soybean plants overexpressing *sHSP26* are more resistant to drought due to lower TBARS levels [42]. In Arabidopsis, *PtCAT2*-overexpressing transgenic lines exhibited significantly lower TBARS content than the wild type under drought stress and enhanced the plant’s drought resistance [43]. Moreover, although proline is widely regarded as a hallmark of plant stress responses, its direct contribution to overall osmotic adjustment is limited due to its relatively low cellular concentration. In sweet sorghum, the contribution of free proline to leaf osmotic potential was reported to be negligible (<0.2%), whereas soluble sugars and betaine were identified as the essential osmotic effectors under saline conditions [44]. Similarly, in transgenic tobacco overexpressing sucrose transporters, enhanced soluble sugar accumulation was directly linked to improved drought and cold tolerance, highlighting the dominant role of sugar metabolism in osmotic adjustment [45,46]. Recent work on *BvKUP13*-overexpressing Arabidopsis further demonstrated that soluble sugars function as key osmotic regulators alongside proline under salt stress [47]. Similarly, our results also found that overexpression of StPIF4 resulted in increased accumulation of both proline and soluble sugars, with the content of soluble sugars being markedly higher than that of proline. These results suggest that *StPIF4* enhances osmotic adjustment capacity, with soluble sugars likely playing a dominant role in maintaining cellular turgor, while proline contributes to membrane stabilization and ROS detoxification.

Leaves function as the principal sites of photosynthesis and are essential for transpiration [48]. Stomatal aperture and leaf relative water loss rate are well-established indirect proxies that reflect transpiration capacity and water conservation status in plants. Stomatal conductance (gs) is the primary physiological determinant of transpiration rate (E), as described by the fundamental transpiration equation incorporating vapor pressure deficit (VPD): E = (VPD/BP) × gs, where BP is the barometric pressure [49]. Under controlled environmental conditions where VPD remains relatively constant, variations in gs directly determine the magnitude of E [48]. Similarly, the rate of water loss from detached leaves, a widely adopted proxy for transpiration rates, has been successfully employed in numerous plant stress physiology studies to evaluate genotypic differences in water conservation capacity [50]. In response to drought stress, plants modify leaf stomatal density and aperture to maintain gas exchange while reducing water loss [51,52,53]. Plant drought tolerance is positively correlated with water retention capacity, where increased retention enhances resistance to drought. Previous studies have shown that overexpression of *StERECTA* in potato reduces stomatal density, thereby improving drought tolerance [54]. Similarly, under drought conditions, overexpression of *HvEPF1* in barley decreases stomatal density, which enhances water-use efficiency and confers greater drought resistance [55]. In apple, overexpression of *MdRaf5* improves drought tolerance by modulating stomatal aperture and transpiration rates [56]. Likewise, in potato, overexpression of *StMAPKK1* promotes stomatal closure, increases antioxidant enzyme activities, and significantly enhances both drought and salt tolerance [57]. In the present study, *StPIF4*-OE plants exhibited reduced stomatal aperture, length, and width under drought stress, along with a significantly lower leaf water loss rate. Collectively, these findings indicate that OE enhances drought tolerance through the modulation of stomatal dynamics.

ABA is a key hormone regulating plant responses to abiotic stress [58]. Research reports indicate that the synthesis and signaling of hormones are influenced by drought stress, and plants cope with drought stress through hormone regulation [59]. For example, ectopic expression of maize *ZmPIF1* and *ZmPIF3* in rice enhanced ABA signaling to induce stomatal closure, significantly reducing transpiration rates and leaf water loss, thereby improving drought tolerance [19]. Similarly, overexpression of *VaNCED1* in grape significantly increased ABA content in transgenic plants, thereby enhancing their drought tolerance [60]. At the same time, studies have found that drought stress increases the significant expression of related genes. In Arabidopsis, after 12 h of PEG treatment, plants overexpressing *PwNAC11* showed significantly higher expression levels of *DREB* compared to the wild type [61]. Plants overexpressing *AtEDT1* showed a significant increase in the expression of the *ABI5* gene [62]. Furthermore, *P5CS* serves as a key rate-limiting enzyme in proline biosynthesis and plays a critical role in maintaining cellular osmotic equilibrium, stabilizing biomembrane integrity, and mitigating oxidative damage triggered by drought or salt stress [63]. As reported by Zhang et al., overexpression of the potato *bZIP* transcription factor *StbZIP1* markedly induced *StP5CS* expression, which consequently led to enhanced proline accumulation and improved saline–alkali tolerance in transgenic plants [64]. These findings are consistent with our research data. This study found that under drought stress, the expression levels of drought-responsive genes (*PGAM*, *DR29A*, *DREB*, *ABI5*, *NCED3*, *P5CS*) and ABA content in OE series plants were significantly higher than those in WT plants (Figure 14). It should be noted that ABA quantification in this study was performed by HPLC with external calibration and spike-recovery monitoring. While extraction efficiency was verified (recovery > 92%), the use of isotope-dilution mass spectrometry with labeled internal standards (e.g., [^2^H_6_] ABA) would provide superior analytical specificity and absolute accuracy, particularly for correcting matrix effects on a per-sample basis. This methodological upgrade will be pursued in follow-up studies to validate the current findings. These findings are consistent with the results reported by Yang et al., who demonstrated that transgenic tobacco overexpressing the potato *StPYL16* gene exhibited significantly elevated endogenous ABA levels under drought stress, accompanied by significant upregulation of drought resistance-related genes [65]. The upregulation of drought-related genes in the overexpression lines of StPIF4 could potentially explain their protective roles in potatoes under drought stress.

## 4. Materials and Methods

### 4.1. Data Sources and Identification of the StPIF Gene Family

The potato reference genome (DM v6.1) was acquired from the Spud Database (Spud DB; http://spuddb.uga.edu/dm_v6_1_download.shtml, accessed on 12 March 2025). Arabidopsis thaliana *PIF* protein sequences were obtained from The Arabidopsis Information Resource (TAIR; https://www.arabidopsis.org/, accessed on 12 March 2025) and utilized as queries in local BLASTP (version 2.16.0, BLAST+ suite; NCBI, Bethesda, MD, USA) searches against the potato proteome, employing an E-value cutoff of ≤1 × 10^−20^. To enhance identification accuracy, the hidden Markov model (HMM) profile of the bHLH domain (PF00010) was downloaded from the Pfam database and applied in HMMER searches. Subsequently, all candidate sequences were validated using the NCBI Conserved Domain Database (CDD) and Pfam to verify the presence of the bHLH domain. After manual removal of redundant sequences, the remaining genes were designated *StPIF1* to *StPIF7* based on their chromosomal distribution.

### 4.2. Phylogenetic Analysis and Classification

Multiple sequence alignment of *StPIF* proteins was conducted using ClustalW (version 1.83) with default parameters. A phylogenetic tree was constructed with MEGA 11 software employing the Maximum Likelihood (ML) method, incorporating 1000 bootstrap replicates. PIF protein sequences from Arabidopsis thaliana, tomato, rice, and maize served as reference sequences for classification. The physicochemical properties of StPIF proteins, including amino acid length, molecular weight (MW), and isoelectric point (pI), were determined using the ExPASy ProtParam tool (http://web.expasy.org/protparam/, accessed on 14 March 2025).

### 4.3. Identification of Gene Structure and Conserved Motifs

The gene structure, specifically the exon–intron organization, of *StPIF* genes was visualized using TBtools, referencing the potato genome annotation. Conserved motifs within *StPIF* proteins were identified utilizing the MEME Suite (http://meme-suite.org/) with the following parameters: maximum number of motifs set to 10, minimum width of 6, and maximum width of 50. The distribution of the identified motifs was subsequently visualized using TBtools.

### 4.4. Gene Duplication of StPIF Genes

Physical location information for *StPIF* genes was sourced from the potato genomic database Spud DB (http://spuddb.uga.edu/dm_v6_1_download.shtml, accessed on 14 March 2025), and all *StPIF* genes were mapped onto the potato chromosomes. The potato reference genome sequence and GFF annotation file were subjected to self-alignment using TBtools to assess the collinearity of *StPIF* members with the Multiple Collinearity Scan toolkit (MCScanX).

### 4.5. Expression Pattern Analysis of the StPIF Gene Family in Different Organs and Under Mannitol-Induced Water Deficit

Using publicly available transcriptome data, we obtained and calculated gene expression levels, which were then normalized to FPKM values (fragments per kilobase of exon per million mapped reads). A heat map depicting gene expression patterns was generated with TBtools software.

### 4.6. Plant Materials and Growth Conditions

The potato cultivar “E3” (wild type, WT), tobacco (*Nicotiana benthamiana* L.), and transgenic potato lines exhibiting *StPIF4* overexpression (OE) and RNA interference (RNAi) served as the experimental materials for this study. Potato and tobacco plants were cultivated in a tissue culture room maintained at 25 °C, following an 8 h dark and 16 h light photoperiod, with a light intensity of 4000 Lux provided by cool white LED lamps. Seedlings of WT, OE, and RNAi lines with consistent growth status were separately transferred to MS solid medium (control, CK) and MS medium containing 150 mM mannitol (drought stress treatment). After 25 days of cultivation, plant height, stem diameter, leaf number, and root morphology indicators (root length, root surface area, root volume, number of root forks) were measured. At the same time, leaves were collected to determine the activities of superoxide dismutase (SOD), peroxidase (POD), catalase (CAT), and the contents of thiobarbituric acid reactive substances (TBARSs), proline, and soluble sugars. Leaf samples were also subjected to DAB and NBT staining to detect H_2_O_2_ accumulation levels, used for scanning electron microscope observation of stomatal morphology and aperture and to measure leaf water loss rate. In addition, leaves were collected, quickly frozen in liquid nitrogen, and stored at −80 °C for RNA extraction and real-time quantitative qPCR analysis of the relative expression levels of *StPIF4* and downstream stress-responsive genes. Each physiological assay included three independent biological replicates. Each biological replicate consisted of three individual plantlets (n = 9 plantlets per treatment per line). For antioxidant enzyme activities (SOD, POD, CAT), TBARSs, proline, soluble sugars, and H_2_O_2_ content, each biological replicate was measured with three technical replicates. For qPCR analysis of gene expression, three biological replicates were performed, each with three technical replicates. For stomatal aperture measurements, 20 stomata were measured per leaf, with three leaves per line per treatment (n = 60 stomata). For leaf relative water content (RWC), three leaves from three independent plantlets were measured per line per treatment.

### 4.7. Cloning and Generation of Transgenic Potato Plants of StPIF4

*StPIF4* overexpression and RNA interference (RNAi) plants were generated by cloning the coding sequence of *StPIF4* and the miRNA interference fragment targeting *StPIF4* into the expression vectors *pC2300s-GFP-35S* and *pART-CAM-RNAi*, respectively. The resulting recombinant plasmids, *pC2300s-StPIF4-GFP* for overexpression and pART-CAM-RNAi-*StPIF4* for RNAi, were then introduced into Agrobacterium tumefaciens strain GV3101. Subsequently, the plasmids were transferred into potato plants (E3) through Agrobacterium tumefaciens-mediated transformation. Positive transgenic lines were identified via PCR using gene-specific primers, followed by quantification of *StPIF4* expression levels in the positive lines using quantitative real-time PCR (qPCR).

A total of 7 independent kanamycin-resistant lines were generated for the overexpression (OE) construct and 5 independent lines for the RNA interference (RNAi) construct, via Agrobacterium tumefaciens-mediated transformation of potato cultivar “E3”. The transformation efficiency was approximately 35% for OE (7 positive lines out of 20 explants) and 25% for RNAi (5 positive lines out of 20 explants). Positive transgenic lines were initially identified by PCR using gene-specific primers and further confirmed by quantitative real-time PCR (qPCR). From the primary positive lines, three independent OE lines (OE-8, OE-10, and OE-11) and three independent RNAi lines (RNAi-5, RNAi-9, and RNAi-11) exhibiting stable inheritance, distinct expression levels, and consistent phenotypes were selected for all subsequent physiological and molecular analyses.

### 4.8. Subcellular Localization of the StPIF4 Gene

The *StPIF4*-GFP primers were designed using the NCBI website (see Table S3). The presence of BamHI and SacI restriction sites in the designed primers was confirmed with Snap Gene software (Snap Gene version 7.1.2). The primers were synthesized by Xi’an Qingke Biotechnology Co., Ltd. (Shanxi, China). The target gene fragment and plasmid vector were digested with the appropriate restriction enzymes and ligated to create the recombinant plasmid, which was then transformed into Escherichia coli for amplification. The verified recombinant plasmid, pC2300300-35S-*StPIF4*-GFP, was subsequently introduced into Agrobacterium tumefaciens strain GV3101. Using a 1 mL needleless syringe, pC2300300-35S-*StPIF4*-GFP was infiltrated into the leaves of uniformly grown, healthy tobacco seedlings. After 3 days of dark incubation, infiltrated leaf tissues were excised, and GFP fluorescence was examined using laser confocal microscopy.

### 4.9. Gene Expression Analysis

Total RNA was extracted from potato plants utilizing the Plant RNA Extraction Kit (Tiangen Biotech, Beijing, China). The RNA concentration was determined with an ultra-micro spectrophotometer (Roche, Basel, Switzerland). Following the manufacturer’s instructions for the TOYOBO (Toyobo Co., Ltd., Osaka, Japan) reverse transcription kit, the RNA was reverse transcribed into complementary DNA (cDNA). Quantitative PCR (qPCR) was conducted using cDNA as the template, employing gene-specific primers to evaluate expression levels. The potato *Actin* gene served as the internal reference gene, and the primers used are listed (Table S2). Data were collected and analyzed through one-way analysis of variance (ANOVA) using Microsoft Excel 2021 and SPSS 22.0 software. The relative expression levels were calculated using the 2^−∆∆CT^ method.

### 4.10. Morphological Characterization of the Transgenic Potato Plants

WT, OE and RNAi potato plants were cultured in vitro on MS agar medium supplemented with either 0 mM or 150 mM mannitol. After 25 days of growth, potato seedlings were subjected to morphological characterization. For each treatment, the number of leaves and plant height were recorded. The fresh and dry weights of the entire plants were measured using an electronic balance. Various root traits, including root length, root number, root volume, number of root tips, fresh root weight, and dry root weight, were assessed in both stressed and unstressed plants. Measurements were conducted using a root scanner (STD 4800; EPSON, Quebec City, QC, Canada) and analyzed with the root image analysis software WinRHIZO version 5.0 (Regent Instruments Inc., Quebec City, QC, Canada).

### 4.11. Determination of Physiological Indicators, Leaf Stomatal Aperture, and Water Loss Rate

Leaves from WT, OE and RNAi transgenic potato plants, cultivated under both normal and drought stress conditions, were collected separately. Staining and observation using 3,3′-diaminobenzidine (DAB) and nitroblue tetrazolium (NBT) were conducted following the methodology outlined by Bi et al. Measurements were taken for stomatal aperture, water loss rate, and the concentrations of osmotic adjustment substances, including proline. The activities of peroxidase (POD), catalase (CAT), and superoxide dismutase (SOD) were assessed using commercial assay kits from Sangon Biotech (Shanghai) Co., Ltd. (No. 698 Xiangmin Road, Songjiang District, Shanghai 201611, China). Additionally, soluble sugar content was determined using the anthrone colorimetric method. Leaf samples (0.1 g) were extracted with 5 mL of distilled water at 100 °C for 30 min. After centrifugation at 8000× *g* for 10 min, 0.2 mL of the supernatant was mixed with 3 mL of anthrone reagent (0.15 g anthrone in 100 mL of 80% H_2_SO_4_) and boiled for 10 min. The absorbance was measured at 620 nm, and glucose was used as a standard. TBARS assay: homogenization of leaf samples (0.5 g) in 0.1% (*w*/*v*) trichloroacetic acid (TCA), centrifugation at 10,000× *g* for 15 min, reaction of supernatant (1 mL) with 0.5% (*w*/*v*) TBA in 20% TCA at 95 °C for 30 min, cooling in an ice bath, and spectrophotometric measurement at 532 nm (corrected at 600 nm). TBARS content was calculated using the molar extinction coefficient of the TBARS-TBA complex (155 mM^−1^ cm^−1^) and expressed as nmol g^−1^ FW. All assays were executed in strict accordance with the manufacturers’ instructions. Each experiment included three independent biological replicates, with each replicate comprising three seedlings.

### 4.12. Determination of ABA Content by HPLC

Leaf samples (0.5 g) were ground into a fine powder in liquid nitrogen and transferred to 15 mL centrifuge tubes. Ten milliliters of pre-cooled 80% methanol (*v*/*v*) containing 0.1 g/L 2,4-dinitrophenol and butylated hydroxytoluene (BHT) were added to each tube. The mixture was sonicated at 22 °C for 2 min, followed by incubation overnight at 4 °C. Following incubation, the extract was centrifuged at 10,000× *g* for 15 min at 4 °C. The supernatant was collected and stored at −20 °C. After that, the aforementioned extraction procedure was repeated once. Following this, the two batches of extracts were combined and concentrated using a high-throughput automatic solid-phase extraction (SPE) system (GOODSPE-8600, Shanghai Kezhe Biochemical Technology Co., Ltd., Room 508, Building 47, No. 2338 Duhui Road, Minhang District, Shanghai 201108, China) and subsequently reconstituted with 1 mL of methanol. Finally, the reconstituted solution was filtered through a 0.22 μm organic phase-compatible filter membrane and stored at −20 °C for subsequent analysis.

The ABA content was determined according to the method described by Wei et al. [26], and the detailed procedures were as follows: For the quantification of ABA, an ABA standard (10 μg/L; Sigma-Aldrich, Cat. No. 90,769−20 mg; HPLC purity ≥ 98%) was employed. Chromatographic analysis was performed using an Agilent 1100 Series high-performance liquid chromatograph (Agilent Technologies, Santa Clara, CA, USA) equipped with a G1314A variable wavelength UV detector (VWD), a G1313A autosampler, and a G1316A column oven. A ZORBAX SB-C18 reversed-phase column (4.6 × 50 mm, 5 μm; Agilent) was employed. The mobile phase was prepared by mixing methanol and 0.6% glacial acetic acid aqueous solution in a 45:55 (*v*/*v*) ratio and degassed using an online degassing unit prior to use. An isocratic elution profile was employed, with a flow rate of 0.8 mL/min, a column temperature maintained at 30 °C, a detection wavelength set to 254 nm, and an injection volume of 20 μL. Under the aforementioned HPLC conditions, ABA displayed a retention time of 8.6 min and a peak area of 12,632.516 m. The retention time for ABA was approximately 8–12 min, and recovery rates ranged from 92.3% to 96.8% (mean ± SD: 94.5% ± 1.8%, n = 6). Method validation demonstrated good linearity for ABA, with a correlation coefficient (R^2^) of 0.9900 over the linear range of 1.0–100 μg/L. Limits of detection (LOD) derived from this method further confirmed that the established protocol exhibits high sensitivity for the determination of endogenous plant ABA.

### 4.13. Data Analysis

All data were initially analyzed using one-way analysis of variance (ANOVA) to assess significant differences among the groups. When ANOVA indicated significant differences, post hoc comparisons were conducted with the least significant difference (LSD) test to elucidate the specific differences between the groups. All statistical tests were performed at significance levels of 0.05 and 0.01, and data analysis and graphing were executed using GraphPad Prism 10 (GraphPad Software Inc., San Diego, CA, USA).

## 5. Conclusions

In this study, seven *StPIF* genes were identified in the potato genome, which were classified into four subfamilies. Among them, the *StPIF4* gene was significantly induced under mannitol-induced water deficit and ABA treatment. We cloned the *StPIF4* gene and generated overexpression and RNAi transgenic lines. Under mannitol-induced water deficit, with WT plants as the control, overexpression of *StPIF4* promoted plant growth and development, enhanced antioxidant enzyme activities, and increased the accumulation of proline, soluble sugars, and ABA, thereby significantly upregulating the expression of drought-responsive genes. Therefore, overexpression of the *StPIF4* gene plays a positive role in potato drought tolerance by reducing stomatal aperture, enhancing reactive oxygen species’ scavenging capacity, and alleviating oxidative damage. These findings enrich the molecular regulatory network of potato drought resistance and may provide valuable approaches for improving potato drought tolerance.

## Data Availability

All data are available within the manuscript.

## Supplementary Materials

The following supporting information can be downloaded at: https://www.mdpi.com/article/10.3390/plants15111623/s1, Figure S1: Genomic distributions of *StPIF* genes on the potato chromosomes; Figure S2: Plasmid map and identification of *StPIF4.* (A) Schematic representation of the construction of the StPIF4 overexpression vector; (B) Schematic representation of the construction of the StJPIF4 RNAi vector. (C) PCR molecular identification of *StPIF4* transgenic lines; Table S1: Information of *StPIF* gene family in potato; Table S2: The sequences of primers were used for qRT-PCR.
