# Supplementary material for: Identification of the StPIFs Gene Family in Potato and Functional Analysis of StPIF4 Under Drought Stress
Source: Plants (Basel). 2026 May 26;15(11):1623. doi: 10.3390/plants15111623 (PMC13259117; doi:10.3390/plants15111623)
Supplement: Supplementary file 1 [file plants-15-01623-s001.zip › Figure S1-S2.pdf]

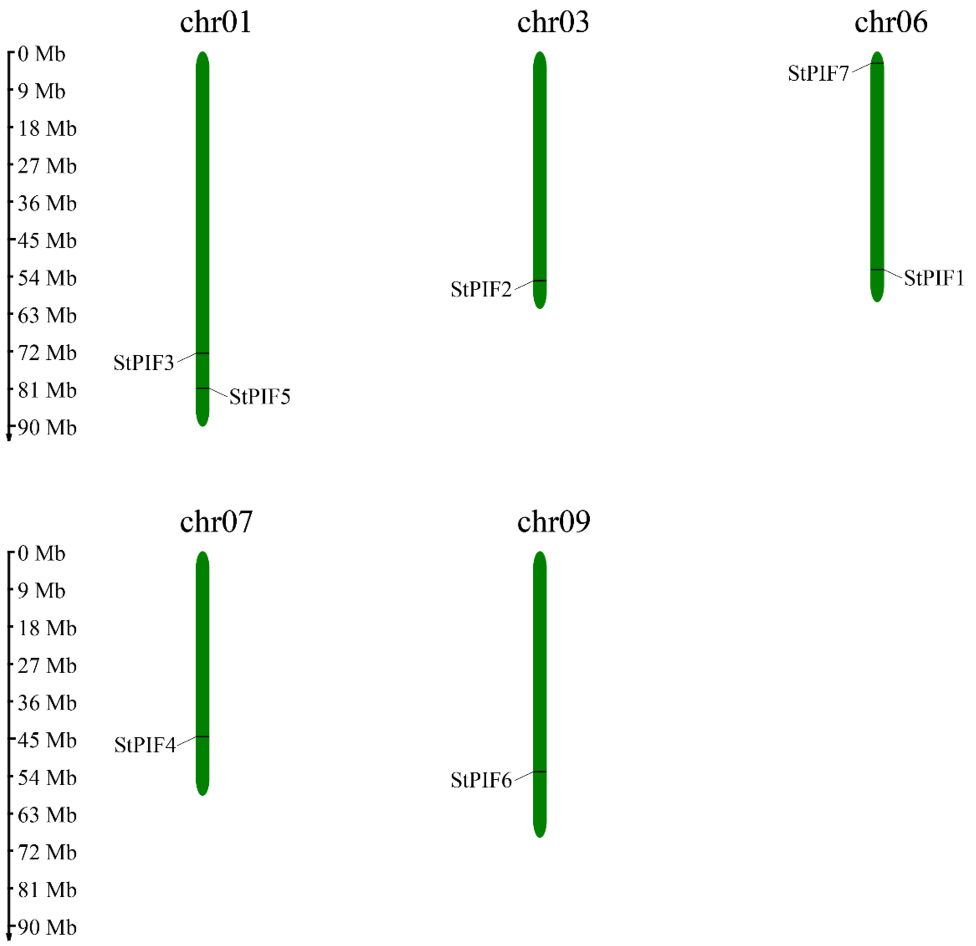

Figure S1. Genomic distributions of *StPIF* genes on the potato chromosomes

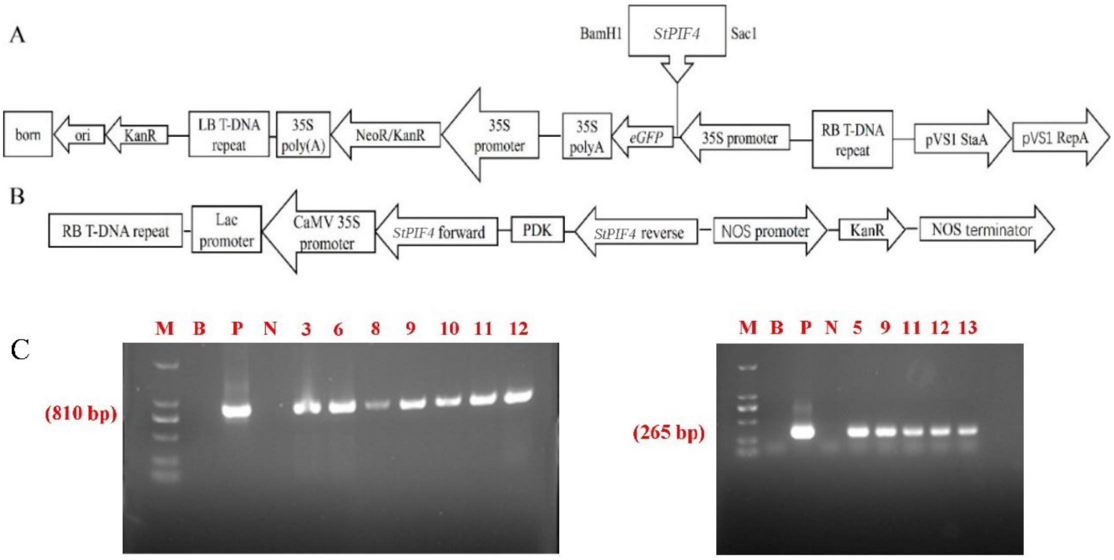

Figure S2. Plasmid map and identification of *StPIF4*. (A) Schematic representation of the construction of the *StPIF4* overexpression vector; (B) Schematic representation of the construction of the *StJPIF4* RNAi vector. (C) PCR molecular identification of *StPIF4* transgenic lines.
